# Breastfeeding Success With Use of Electric Breast Pump Versus Inverted Syringe Technique in Lactating Women With Inverted Nipple: Open Labelled Randomized Control Trial

**DOI:** 10.7759/cureus.68153

**Published:** 2024-08-29

**Authors:** Bhabesh K Chowdhry, Anagha Nair, Sangam Jha, Amrita Banerjee, Pallavi Singh, Richie Dalai

**Affiliations:** 1 Neonatology, All India Institute of Medical Sciences, Patna, IND; 2 Pediatrics, All India Institute of Medical Sciences, Patna, IND; 3 Obstetrics and Gynaecology, All India Institute of Medical Sciences, Patna, IND; 4 Paediatrics, All India Institute of Medical Sciences, Patna, IND

**Keywords:** nipple pain, breastfeeding women, electric breast pump, syringing, inverted nipple

## Abstract

Background

Inverted nipple is a commonly encountered impediment to proper attachment and latch establishment. Correction of inversion using a disposable syringe represents the conventional method of management. However, it is understudied, cumbersome, and inconvenient. Using electric breast pump represents a more physiological method to achieve correction of inversion. This open-label, randomized control trial investigated syringing versus electric breast pump both in terms of effectiveness in achieving correction of inversion of nipple and patient satisfaction with the use of the assigned intervention.

Objectives

The primary objective of this study was to compare breastfeeding success by Day 3 postnatal age (achieving correction of inversion of nipple and establishment of direct breastfeeding) in syringing versus electric breast pump in mothers with inverted or flat nipples. The secondary objective was to compare the two methods for the pain experienced by the mother while using syringing versus an electric breast pump.

Methodology/Design

This was a single-center, open-label randomized control trial performed at a tertiary care neonatal unit in Eastern India, from December 2022 to September 2023. 60 healthy mothers with inverted nipples were randomly allocated to one of two interventions: Group A (n=30, syringing); or Group B (n=30, electric breast pump). Both groups were compared for the establishment of breastfeeding by Day 3 postnatal age and daily maximum pain scores till Day 3 (visual analogue scale). Multivariable logistic regression was used to look for factors independently associated with establishment of breast feeding by Day 3 postnatal period. Chi square test was used to compare the proportions of different outcomes between the groups.

Results

The primary outcome of establishment of breastfeeding by Day 3 postnatal period was achieved in 18 (60%) mothers in syringe group vs. 17 (56.67%) in the breast pump group, with no statistically significant difference (p=0.793). In the first two days the pain score differences were not statistically significant, but on Day 3, 28 (93.99%) mothers in the electric breast pump group had no/mild pain compared to 22 (73.33%) mothers in the syringe usage group. This difference was statistically significant (p= 0.038).

Conclusion

In hospital settings where electric breast pump is easily available, the same may be preferred over inverted syringe technique for achieving establishment of breastfeeding by Day 3 postnatal period with minimal nipple pain in mothers. However, further large scale studies will be required to confirm these findings.

## Introduction

Exclusive breastfeeding is essential for the improved survival of neonates and enhances their nutritional and immunological parameters [1]. Despite best efforts, some factors create impediments to the establishment of successful breastfeeding. One prominent factor among them is the inversion of the nipple which leads to difficult latching and attachment. Inversion of the nipple can be congenital or acquired. The congenital form has an estimated prevalence of 3-10% in the general population [2]. This remains asymptomatic until the requirement to perform breastfeeding arises. It occurs due to a misstep in the normal embryological development. Normally proliferation of mesoderm ensures that the nipple rises above the areola [3]. Failure of this to occur results in an inverted nipple. Acquired inversion is usually secondary to mastitis, breast malignancy, and surgery. Acquired causes are much rarer than congenital forms but require detailed evaluation due to the sinister possibilities behind their occurrence [4]. Management of this condition depends on the grade of inversion. Han and Hong have classified inverted nipples into three grades: Grade 1 (minimal fibrosis, nipple can be easily pulled out, maintains projection); Grade 2 (moderate fibrosis, nipple can be pulled out manually, fails to maintain projection); and Grade 3 (severe fibrosis, requires surgical correction as it cannot be pulled out manually) [5].

Non-surgical methods that have been employed for Grades 1 and 2 inverted nipples include Hoffman exercises, Woolwich breast shield, Niplette^TM^, and encircling the base of the nipple with a rubber band. Of these former two did not have any significant effect on the correction of inversion whereas the latter two had a 100% success rate in the establishment of direct breastfeeding [4]. This percentage is based on a study of 22 women who used Niplette^TM^ and 19 women who used the rubber band. Thus, further studies with larger sample sizes are required to establish available findings. The non-surgical method that gained maximum traction for the management of inverted nipples was however the use of a disposable 10 mL syringe which was first introduced by Kesaree et al. in 1993 [6]. Despite its popular use in clinical practice, the first randomized control trial to assess its efficacy was conducted in 2018 by Nabulsi et al., whose study reported that there was no improvement in rates of breastfeeding up to six months postpartum, quality of life and breastfeeding satisfaction with use of disposable syringe [4].

The past two decades have witnessed a rise in the use of breast pumps to express breast milk. Improvements in scientific advances and the betterment of socioeconomic status have allowed for easy access to electric breast pumps. Its use has become rather popular among the new echelon of working women who proactively use this device to ensure that their babies have access to the best form of nutrition even during periods of their absence. Breast pumps employ a suction effect to bring about the expression of milk, which is very similar to the suction produced by an infant suckling at the breast [7]. In comparison to suction created by a disposable syringe, that created by an electric breast pump is more uniform, sustained, and physiological. To the best of our knowledge, no study has been conducted to compare the use of disposable syringes with electric breast pumps in women with inverted nipples. In this study, we aim to address precisely this knowledge gap and attempt to establish that an electric breast pump is comparable to syringing in the establishment of breastfeeding in women with an inverted nipple.

## Materials and methods

Study design

This was an open-label, single-center, randomized control trial conducted over 10 months, from December 2022 to September 2023. It was approved by the Institutional Research Committee and Institutional Ethics Committee (Reference number AIIMS/Pat/IEC/2022/956). Trial registration was done under the International Standard Randomised Controlled Trial Number (ISRCTN) registry with registration number ISRCTN17899692. Written informed consent was obtained from all participants before enrollment and randomization.

Study participants

Mothers who presented to the center for delivery were approached for enrolment in the study. Inclusion criteria was lactating mothers with inverted nipples with neonates born at term gestation (>37 weeks period of gestation), and APGAR>7/10 at birth. Mothers with breast cancer or on cytotoxic/chemotherapy drugs and those requiring admission in the ICU within 24 hours of delivery were excluded. Mothers having neonates requiring neonatal ICU (NICU) admission within 24 hours of birth or having oro-pharyngeal abnormalities like cleft lip/palate were also excluded. Mothers fitting the inclusion criteria were identified either in the antenatal period or immediate postpartum period during the attempt to initiate direct breastfeeding and were intimated to the research team.

Randomization

Within 24 hours of the birth of the baby, the participant was randomly allocated to one of two intervention arms (Group A: Syringing group; Group B: Electric breast pump group) in a 1:1 ratio. The random sequence was computer-generated and block randomization with blocks of variable sizes was done. This sequence was concealed using sequentially numbered opaque sealed envelopes. The participant’s allocation was revealed only after obtaining written informed consent.

Intervention

**Figure 1:**
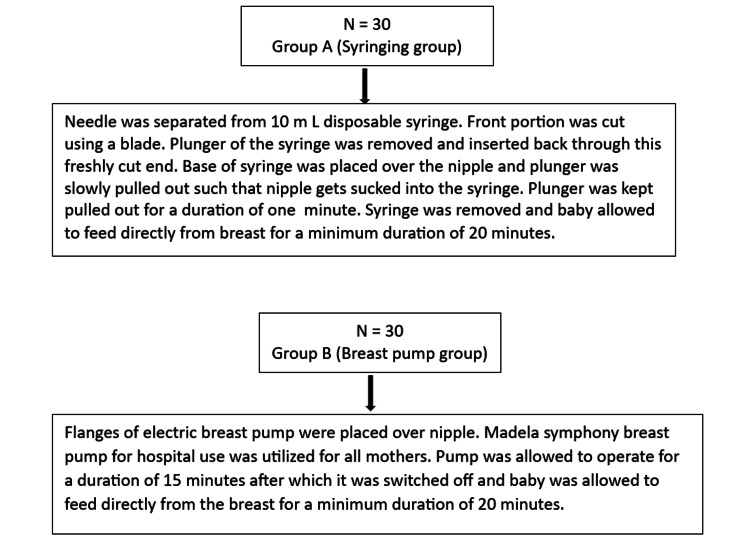
Interventions performed for inverted nipple in comparison groups.

Mothers allocated to Group A were instructed to use a cut, disposable 10 mL syringe to evert their nipple before each feed. Mothers were shown how to place the cut syringe over their nipple and gently pull till the nipple was everted, maintaining it for one minute, after which the syringe was removed and breastfeeding started. They were asked to feed for a minimum duration of 20 minutes. Mothers allocated to Group B were instructed to apply the Madela Symphony electric breast pump (which was made available in their respective postnatal ward) to their breasts for 15 minutes or longer if eversion was not yet achieved. Following this they were instructed to latch the baby on breast and feed for a minimum duration of 20 minutes (Figure 1).

Mothers in both groups were asked to carry out this process till either eversion of the nipple was maintained in the period in between feeding for four consecutive feeds or till the third postpartum day, whichever was earlier.

Outcome measures

The primary outcome measured was whether direct breastfeeding could be established by Day 3 postpartum period without the need for continued intervention to evert the nipple. Secondary outcome measures included the number of times intervention was utilized per day and the degree of pain experienced by the mother after using the assigned intervention as assessed by visual analog pain scale with no 0 score representing no pain, a score of 1-3 representing mild pain, and a score of >3 representing moderate to severe pain.

Data collection

Baseline data was collected for all participants and included maternal age, period of gestation at birth, and the grade of inverted nipple. Rate of successful latching and attachment with satisfactory breastfeeding was checked at Day 3 of study and recorded. All participants were also asked to note if there was pain after the use of the intervention, at the end of each day and the maximum severity was documented using a visual analog pain scale in each group. This data was noted either till the day up to which intervention was used or till the third postpartum day, whichever was earlier. The collected data was entered into a Microsoft Excel sheet.

Sample size

A convenient sample size of 60 was taken, with 1:1 allocation ratio among the two intervention arms. This was to mainly look at the feasibility of the interventions at a hospital, in a low-middle income country. 

Statistical analysis

All analysis was done using STATA 18/BE (Stata Corp statistical package version BE/18.0). Descriptive analyses were done to describe the maternal characteristics like age, grade of inverted nipple, and birth details like gestational age. The continuous variables were first checked for normality and accordingly presented as mean (SD) or median (interquartile range (IQR)). The categorical variables were expressed as n (%). Multivariable logistic regression was used to look for factors independently associated with establishment of breast feeding by Day 3 postnatal period. Chi square test was used to compare the proportions of different outcomes between the groups.

## Results

**Figure 2:**
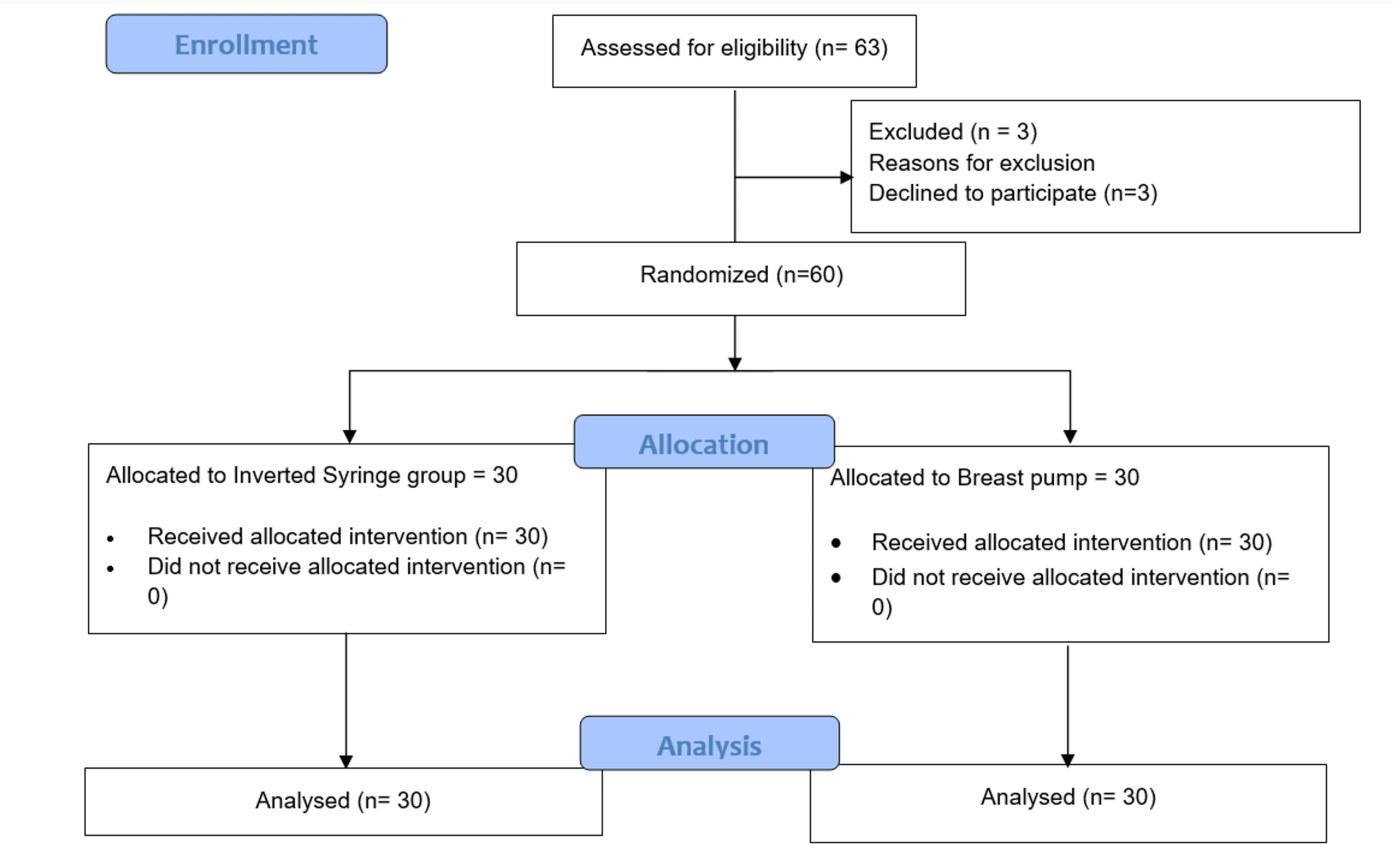
Study flow diagram

A total of 63 mothers were found eligible over the study period of 10 months, of which three declined consent for participation. The remaining 60 mothers were enrolled, with 30 mothers undergoing intervention for inverted nipples with syringing (Group A) and 30 mothers undergoing intervention for inverted nipples with an electric breast pump (Group B) (Figure 2). Of these mothers, the maternal age, the gestational age at birth and the grade of inverted nipple were similar in both groups (Table 1). 19 (63.33%) mothers in the inverted syringe group and 20 (66.67%) in the electric breast pump group were primigravida. This difference was not statistically significant (p-value=0.787).

**Table 1:** Baseline characteristics of mothers enrolled in the study *Expressed as Mean (SD); †Expressed as n (%) LSCS: Lower segment cesarean section

Baseline characteristics	Group A (n= 30)	Group B (n=30)	n=60
Age of mothers (years)*	25.6 (4.3)	24.9 (3)	25.2 (3.7)
Mode of delivery†			
Vaginal delivery	6 (20)	11 (36.67)	17 (28.3)
LSCS (Emergency)	16 (53.33)	15 (50)	31 (51.7)
LSCS (Elective)	8 (26.67)	4 (13.33)	12 (20)
Gestational weeks at birth*	38.4 (1.2)	38.3 (1.7)	38.3 (1.5)
Grade of inverted nipple†			
Grade 1	7 (23.33)	6 (20)	13 (21.7)
Grade 2	21 (70)	22 (73.33)	43 (71.7)
Grade 3	2 (6.67)	2 (6.67)	4 (6.6)

 The primary outcome of establishment of breastfeeding by Day 3 postnatal period was achieved in 18 (60%) mothers in syringe group vs. 17 (56.67%) in the breast pump group, with no statistically significant difference (p=0.793). The factors associated with establishment of breastfeeding by Day 3 were explored with multivariable logistic regression. Though there was a trend towards higher odds of establishment of breastfeeding by Day 3 with greater maternal age and gestational age at birth, this association was not found to be statistically significant (Table 2). Moreover, these factors as already mentioned were equally distributed in both the intervention groups.

**Table 2:** Multivariable logistic regression analysis for establishment of breastfeeding by Day 3 for association with maternal age, gestational age at birth, and the grade of inverted nipple

Independent variable	Odds ratio	95% CI	p-value
Age of mother	1.1	0.94-1.28	0.215
Completed gestational age	1.35	0.93-1.97	0.116
Grade of inverted nipple	0.745	0.25-2.19	0.593

The secondary outcome of pain was assessed in mothers daily and the maximum pain experienced was documented with scoring as per the visual analogue scale. In both groups the pain scores gradually decreased from Day 1 to Day 3 postnatal age (Figure 2). The comparison of both groups for the proportion of mothers with severe pain showed that, on Days 1 and 2, there were not statistically significant differences. However, on Day 3, the breast pump group had significantly lower proportion of mothers with severe pain compared to those using syringe technique (p=0.044) (Table 3).

**Figure 3:**
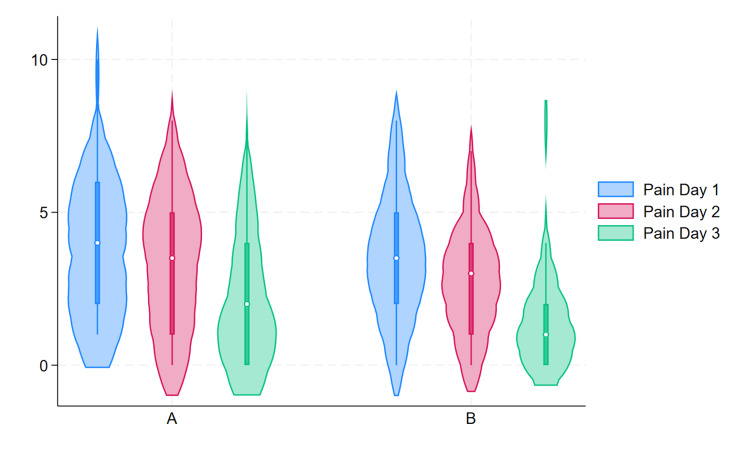
Vioplot of pain scores from Day 1 to Day 3 postnatal period in Group A vs. Group B

**Table 3:** Comparison of outcome variables between the two groups *n (%); †Severe pain on visual analogue scale defined as score≥5

Outcome variable*	Group A (Syringe group) (n=30)	Group B (Breast Pump group) (n=30)	p-value
Establishment of breastfeeding by Day 3	18 (60)	17 (56.67)	0.793
Severe pain† on Day 1	13 (43.33)	8 (26.67)	0.176
Severe pain† on Day 2	10 (33.33)	5 (16.67)	0.136
Severe pain† on Day 3	6 (20)	1 (3.33)	0.044

 Also the pain profile of all mothers was assessed further, comparing the two groups for the proportion of mothers with no, mild and moderate to severe pain. In both groups the proportion of mothers with no/mild pain increased with the postnatal days. In the first two days the difference was not statistically significant, but on Day 3, 28 (93.99%) mothers in the breast pump group had no/mild pain compared to 22 (73.33%) mothers in the syringe usage group. This difference was statistically significant (p= 0.038) (Table 4). There were no sore/cracked nipple reported by the mothers after usage of either interventions.

**Table 4:** Pain profile of lactating mothers using syringing (Group A) vs. electric breast pump (Group B) on first three days of postnatal period *n (%)

Day of postnatal period	Severity of pain*	Group A (n=30)	Group B (n=30)	p-value
1	No or mild pain	14 (46.67)	15 (50)	0.796
	Moderate to severe pain	16 (53.33)	15 (50)	
2	No or mild pain	15 (50)	21 (70)	0.114
	Moderate to severe pain	15 (50)	9 (30)	
3	No or mild pain	22 (73.33)	28 (93.33)	0.038
	Moderate to severe pain	8 (26.67)	2 (6.67)	

## Discussion

Few studies have been done where the efficacy of the inverted syringe technique has been assessed in mothers with inverted nipples after its introduction by Kesaree et al. in 1993 [6]. Bagal et al. compared the usage of the inverted syringe technique in the antenatal period in 30 pregnant women with inverted nipples versus standard care. They found similar breastfeeding success and mean weights of neonates born to mothers in both groups [8]. Nabusi et al. conducted a pilot randomized trial on 45 pregnant individuals comparing inverted syringe technique with standard care. The improvement in nipple inversion was similar between both groups. The complications associated with breastfeeding were also comparable at a one-month follow-up period but were more in the standard group at a three-month follow-up period [9]. In these studies, inverted syringe technique was compared with standard care. 

Although there have been studies comparing other individual methods (Hoffman's technique, rubber band method, Inverted syringe technique) with standard care (control), there are no trials studying the efficacy of usage of electric breast pump in breastfeeding success in mothers with flat nipple. A recent systematic review of studies comparing these interventions (Hoffman’s technique, syringe technique, and nipple exercises) with standard care, found that all the interventions were effective in correction of flat or inverted nipple. However, superiority of one intervention over the other couldn't be proved and they concluded that further studies are required regarding the same [10].

In our study, we compared two different interventions namely the inverted syringe technique and an electric breast pump for inverted nipple in lactating mothers, in parallel groups to see if there was a significant difference between these interventions, in terms of establishment of breast feeding by Day 3 postnatal period and nipple pain felt by the lactating mothers. Our study is the first such randomized controlled trial, with a novel research question and robust methodology, comparing these two techniques for inverted nipple in lactating mothers. All previous randomized trials have focused on single interventions versus standard care. Both the methods in our study led to a similar proportion of mothers with established breastfeeding by Day 3 postnatal period. The pain in the nipple experienced by the mothers was also similar in both groups in the initial 48 hours. But the electric breast pump group had a significantly higher proportion of mothers with no/mild pain after 48 hours of the postnatal period.

There were certain limitations in our study. The study was done at a single center. Also, the study had a small sample size of 60. This was taken to look at the feasibility of the interventions at a tertiary care setting in a low-middle income country. In this study, even though the proportion of mothers with severe pain in nipple after 48 hours was significantly lesser in the electric breast pump group, further studies with a larger sample size are needed to do the cost-benefit analysis. Moreover, an electric breast pump may not be easily available at all centers in low-middle-income countries. Hence, the findings in this study may not be generalizable to all neonatal centers in low-middle-income countries.

The other non-invasive methods of interventions that have been studied, to correct inverted nipple include Hoffman’s exercises, inverted syringe method, rubber band technique, and conventional physiotherapy. The Hoffman’s technique refers to nipple stretching exercises first defined by Hoffman in 1953. These are done by placing the nipple between the thumb and index fingers and by taking the fingers off the areola slowly, repeating five times horizontally and five times vertically [11-13]. The rubber band technique involves injector initiated rubber band placement at the base of the nipple which helps maintain eversion in the nipple after application of inverted syringe [14,15]. The conventional physiotherapy method involves hot fomentation of the breast followed by kneading for 15 minutes [12]. Further studies comparing these other methods with each other are also required.

## Conclusions

Since both the methods in our study led to a similar proportion of mothers with established breastfeeding by Day 3 postnatal period, with the electric breast pump group having a significantly higher proportion of mothers with no/mild pain after 48 hours of the postnatal period, the authors conclude that in hospital settings, where an electric breast pump is easily available, the same may be preferred over an inverted syringe technique for achieving the establishment of breastfeeding by Day 3 postnatal period with minimal nipple pain in mothers. However, caution should be exercised in generalizing these findings to the settings with no/inadequate number of electric breast pumps available compared to the number of admitted mothers with inverted nipples. In such a scenario, since the inverted syringe technique has been found to have comparable efficacy in establishment of breastfeeding by Day 3 postnatal period, the same may be used.

Further large-scale trials in low-middle-income countries will be required, to compare these two methods, to find if a difference in efficacy of establishment of breastfeeding truly exists and if using an electric breast pump in a hospital setting is cost-effective.
